# Thrombocytopenia in Dengue: Interrelationship between Virus and the Imbalance between Coagulation and Fibrinolysis and Inflammatory Mediators

**DOI:** 10.1155/2015/313842

**Published:** 2015-04-27

**Authors:** Elzinandes Leal de Azeredo, Robson Q. Monteiro, Luzia Maria de-Oliveira Pinto

**Affiliations:** ^1^Laboratório de Imunologia Viral, IOC, FIOCRUZ, Avenida Brasil 4365, Manguinhos, 21040-360 Rio de Janeiro, RJ, Brazil; ^2^Instituto de Bioquimica Médica Leopoldo de Meis, Universidade Federal do Rio de Janeiro, 21941-599 Rio de Janeiro, RJ, Brazil

## Abstract

Dengue is an infectious disease caused by dengue virus (DENV). In general, dengue is a self-limiting acute febrile illness followed by a phase of critical defervescence, in which patients may improve or progress to a severe form. Severe illness is characterized by hemodynamic disturbances, increased vascular permeability, hypovolemia, hypotension, and shock. Thrombocytopenia and platelet dysfunction are common in both cases and are related to the clinical outcome. Different mechanisms have been hypothesized to explain DENV-associated thrombocytopenia, including the suppression of bone marrow and the peripheral destruction of platelets. Studies have shown DENV-infected hematopoietic progenitors or bone marrow stromal cells. Moreover, anti-platelet antibodies would be involved in peripheral platelet destruction as platelets interact with endothelial cells, immune cells, and/or DENV. It is not yet clear whether platelets play a role in the viral spread. Here, we focus on the mechanisms of thrombocytopenia and platelet dysfunction in DENV infection. Because platelets participate in the inflammatory and immune response by promoting cytokine, chemokine, and inflammatory mediator secretion, their relevance as “immune-like effector cells” will be discussed. Finally, an implication for platelets in plasma leakage will be also regarded, as thrombocytopenia is associated with clinical outcome and higher mortality.

## 1. Dengue: General Aspects

Dengue viruses (DENVs) are the most important human arboviruses worldwide and are transmitted by mosquitoes of the genus* Aedes* in the form of four distinct serotypes (DENV-1, DENV-2, DENV-3, and DENV-4). Dengue causes serious infection in humans, resulting in morbidity and mortality in most tropical and subtropical areas of the world. It is estimated that there are currently 50–100 million cases of dengue every year worldwide, including more than 500,000 reported cases of dengue hemorrhagic fever and dengue shock syndrome (DHF/DSS) [1]. DENVs are members of the Flaviviridae family, which are single-stranded RNA viruses of positive polarity with approximately 11,000 nucleotides and one phase open reading frame that encodes a single polyprotein, which is subsequently cleaved into three structural proteins (C, prM/M and E) and seven nonstructural proteins (NS1, NS2A, NS2b, NS3, NS4A, NS4B, and NS5) [2, 3]. The structural proteins include a capsid protein (C) that binds viral RNA [4], a Membrane protein (M) found in the mature viral particle, and an envelope (E) protein that mediates viral attachment, membrane fusion, and virion assembly [5]. The E protein is the major structural protein exposed on the surface of the viral particle that triggers protective immune responses in the host by eliciting the production of neutralizing antibodies. The E protein is composed of 3 domains; domain I contains the central region, domain II is involved in virus-mediated membrane fusion, and domain III interacts with cell receptors and contains epitopes recognized by neutralizing antibodies [6].

The nonstructural proteins are involved in viral translation, transcription, and replication. NS1 is a 46 kDa protein involved in viral RNA replication. Notably, NS1 is expressed on the surface of infected cells without forming part of the virion [7]. Serum levels of secreted NS1 (sNS1) positively correlate with viral titers and have been a useful tool in dengue infection diagnosis [8, 9]. Because it is expressed on the surface of infected cells, NS1 triggers host immune responses. Additionally, NS1 has been shown to display soluble complement-fixing activity [10], and it was suggested to be involved in dengue pathogenesis [11]. NS2A is a 22 kDa protein involved in RNA packaging and replication, and it may be involved in interferon type I antagonism [12, 13]. NS2B is a 14 kDa membrane-associated protein and serves as a cofactor for NS3 to form a viral protease complex [14, 15]. NS3 is a multifunctional protein with serine protease helicase/nucleoside triphosphate-NTPase activities, and it is required for unwinding the double-stranded replicative form of RNA. It is also involved in processing the viral polyprotein and RNA replication [7, 16]. NS4A and NS4B are small hydrophobic proteins that function as IFN-signaling inhibitors [12, 13]. Finally, NS5 is a large multifunctional 103 kDa protein that displays RNA-dependent polymerase activity and was recently identified as a potential type I IFN production antagonist [17, 18].

All dengue serotypes are capable of causing disease with a wide spectrum of clinical manifestations, ranging from an undifferentiated fever in a mild clinical form classically known as dengue fever (DF) to the severe clinical and potentially fatal DHF/DSS [19]. Initial symptoms are common to all patients, but the clinical manifestations of the severe forms rapidly evolve with symptoms including high fever, liver enlargement, circulatory failure (hypotension and shock), edema cavity (pleural, abdominal, and cardiac) and internal bleeding phenomena. The severe forms are primarily characterized by plasma leakage and thrombocytopenia with or without hemorrhage. The World Health Organization (WHO) classified the clinical presentations of DHF into four severity grades based on laboratory data: Grade I: fever with positive tourniquet test; Grade II: plus mild spontaneous bleeding; Grade III: presence of weak and rapid pulse; and Grade IV: profound shock with undetectable pulse. The last two are considered DSS [20].

It has been difficult to apply the WHO classifications in Central American countries and Latin America [21–23], suggesting that the disease classification into DF, DHF, and DSS may not be universally applicable for clinical management. In this context, Harris et al. (2000) reported several clinical DSS cases that could not be completely classified as above. Given the difficulty of classifying serious cases according to WHO criteria, a new category has been proposed called* Signs Associated with Dengue Shock*, which is very similar to DSS but without the presence of thrombocytopenia and hemoconcentration [22]. The Special Programme for Research and Training in Tropical Diseases World Health Organization (TDR/WHO) proposed a new classification based on the difficulties encountered in applying the criteria of the WHO classification, especially in Latin America. The classification is based on current clinical criteria for severity of dengue cases, understanding of the illness as a systemic and dynamic event, and facilitating the approach to cases and subsequent clinical management of patients. According to this criterion, the new classification addresses three sets of clinical signs and symptoms: (1) dengue without warning signs, (2) dengue with warning signs, and (3) severe dengue [24, 25]. According to the new classification, patients typically developed a sudden high-grade fever. This acute febrile phase usually lasts 2–7 days and was often accompanied by facial flushing, skin erythema, generalized body ache, myalgia, arthralgia, and headache. Anorexia, nausea, and vomiting were also common. These clinical features are characterized as nonsevere dengue cases. Monitoring for warning signs and other clinical parameters are crucial to recognizing the progression to the critical phase, and it may help distinguish nonsevere and severe dengue cases. The warning signs were assessed by the following clinical parameters: abdominal pain or tenderness, persistent vomiting, clinical fluid accumulation, mucosal bleed, lethargy, restlessness, and liver enlargement >2 cm associated with laboratory parameters such as increase in hematocrit (HCT) concurrent with rapid decrease in platelet count. Mild hemorrhagic manifestations such as petechiae and mucosal membrane bleeding (e.g., nose and gums) may be present. Patients require emergency treatment and urgent referral in the critical phase of disease, that is, when they have severe dengue, characterized by severe plasma leakage leading to dengue shock and/or fluid accumulation with respiratory distress, severe hemorrhages shown by massive vaginal bleeding (in women of childbearing age), and gastrointestinal bleeding and/or severe organ impairment (hepatic damage, renal impairment, cardiomyopathy, encephalopathy, or encephalitis) [25]. Most patients recovered from the illness uneventfully and survived to hospital discharge.

The mechanisms by which pathophysiologic changes occur in dengue fever are still not fully understood. The interaction of several factors seems to be responsible for the development of the severe disease. These factors include the following: the virulence of the circulating strain, the presence of efficient or high density vector, the wide circulation of the virus, and characteristics of the host as genetic factors, ethnicity, presence of chronic diseases, and subsequent DENV infections [19, 26]. Natural infection by one of the four DENV serotypes produces lasting immunity against reinfection by the same serotype, but heterotypic protection is temporary and partial, thus allowing sequential infections. Epidemiological studies have shown that most individuals who develop the more severe DHF/DSS had been previously infected with a different serotype. These observations indicate that a previous infection with dengue is a risk factor for development of severe illness. The antibody-dependent enhancement (ADE) mechanism was proposed by Halstead in the 1970s [27].

One striking finding that potentially plays a critical role in dengue immunopathogenesis occurs during sequential infections by heterologous DENV serotypes. The phenomenon known as the* Original Antigenic Sin* was first described in relation to influenza responses involving B cells [28], and, more recently, in humans infected with DENV [29]. According to the phenomenon of* Original Antigenic Sin*, T cells generated during primary infection with a viral serotype showed a low affinity for the serotype of the second infection, which in turn leads to development of altered immune responses, including decreased viral clearance. In addition, the extensive activation of cross-reactive memory cells promotes the aberrant release of cytokines (cytokine storm) that contribute to disease severity [30].

## 2. Thrombocytopenia in Dengue

Thrombocytopenia has always been one of the criteria used by WHO guidelines as a potential indicator of clinical severity [20, 31]. In the most recent 2009 WHO guidelines, the definitions generally describe a rapid decline in platelet count or a platelet count less than 150,000 per microliter of blood [24].

A kinetic description of platelet count in DHF/DF showed a significant decrease on the 4th day of the illness. In fact, previous studies reported DHF in adults without shock, in which platelet counts mildly to moderately decreased on the 3rd day until the 7th day of illness and reached normal levels on the 8th or 9th day [32–34]. In children, there is little correlation between platelet count and bleeding manifestations or between platelet count and disease severity [35, 36]. In adults, a platelet count of 5 × 10^9^ L^−1^ and packed cell volume >50 are significantly associated with bleeding manifestations. However, a study enrolling 245 dengue patients showed no correlation between clinical bleeding and platelet count, and 81 nonbleeding patients had counts of less than 20 × 10^9^ L^−1^ [37]. In contrast, another study enrolling 225 dengue patients suggested that bleeding occurred more often in patients with platelet counts below 20 × 10^9^ L^−1^ [38].

Most clinical guidelines recommend that platelet transfusions be given to patients who develop serious hemorrhagic manifestations or have very low platelet counts, platelet counts falling below 10–20 × 10^9^ L^−1^ without hemorrhage or 50 × 10^9^ L^−1^ with bleeding or hemorrhage. The efficacy of platelet transfusions is controversial. In a study of 106 pediatric patients with DSS with thrombocytopenia and coagulopathy, there was no significant difference in hemorrhage between patients who received preventive transfusions compared to those who did not. Patients who received transfusion had a higher frequency of pulmonary edema and increased length of hospitalization [39]. Platelet transfusion did not prevent the development of severe bleeding or shorten the time to bleeding cessation and was associated with significant side effects. Thus, according to the authors, platelet transfusions should not be routinely performed in the management of dengue [40, 41].

The mechanisms involved in thrombocytopenia and bleeding during DENV infection are not fully understood. Several hypotheses have been suggested to elucidate the mechanism involved. In this context, DENV could directly or indirectly affect bone marrow progenitor cells by inhibiting their function [42] to reduce the proliferative capacity of hematopoietic cells [43]. Indeed, there is evidence that DENV can induce bone marrow hypoplasia during the acute phase of the disease [44]. Besides platelets counts, the functional disruption of these cells is associated with a significant deregulation of the plasma kinin system and the immunopathogenesis of dengue [45]. In addition, DENV infection induces platelet consumption due to disseminated intravascular coagulation (DIC), platelet destruction due to increased apoptosis, lysis by the complement system and by the involvement of antiplatelet antibodies [46–48]. Here, we discuss the relevance of platelets in physiology and their implication in dengue pathogenesis, acting both as a* victim* of infection and an* effector cell* of the antiviral immune response.

## 3. Platelets: General Aspects

Platelets are the cellular effectors of primary hemostasis, as they contribute to thrombus formation at sites of vascular injury, a fundamental tenet in physiology and medicine [49–55]. Platelets are anucleated circulating cells in mammals approximately 2 *μ*m in diameter and are derived from megakaryocytes within the bone marrow [56, 57]. There are approximately 10^12^ platelets circulating in the blood of an adult human, and, because the lifespan of an individual platelet is only 8–10 days, 10^11^ new platelets must be produced daily from bone marrow megakaryocytes to maintain normal platelet counts (150–400 × 10^9^ platelets per liter of blood) [58].

The resting platelet plasma membrane is generally smooth, except for periodic invaginations delineating the entrances to the open canalicular system and a system of folded membranes. The canalicular system consists of a complex network of intertwining membrane tubes that permeate the platelet's cytoplasm. Meanwhile, the folded membrane systems allow the platelets to have a large surface area and readily take up proteins and molecules and re-release them upon activation. The lentiform shape of the resting platelet is maintained by three major cytoskeletal components: the marginal microtubule coil, the spectrin-based membrane skeleton, and the actin-based cytoskeleton [59]. When platelets encounter a damaged vessel wall, they become activated and undergo a dramatic actin-mediated shape change from smooth discoid to spiny spheres. This process is initiated by a Ca^+2^ influx, which promotes the formation of finger-like filopodia and pseudopods. During this reaction, the number of receptors on the platelet membrane for adhesive and clotting proteins increases, and activated platelets attract other platelets, which clump together and ultimately form a plug that seals the vascular leak [60].

At the resting state, the platelet membrane is virtually impermeable to Ca^+2^. Phosphatidylserine (PS) is a phospholipid in the internal hemilayer of the platelet membrane at rest, and it is the main determinant of platelet procoagulant activity. When platelets are activated, PS is exposed on the external hemilayer, which is usually associated with the formation of platelet-derived microparticles (PMPs), which also have prothrombinase activity [61]. Changes in intraplatelet Ca^+2^ concentration as a result of Ca^+2^ influx or mobilization of intracellular stores are fundamental to the platelet activation response and precede several activation responses, such as shape change, aggregation, secretion, and expression of procoagulant activity. In this process, P-selectin (CD62P) translocates from the *α*-granule membrane to the platelet membrane, where it contributes to platelet-leukocyte, platelet-endothelium, and platelet-monocyte binding and thromboembolic tendency [62]. In addition, activated platelets release soluble CD154, also called CD40 ligand or CD40L, which can interact with vascular cells (including endothelial cells) and induce E-selectin (CD62E) and CD62P upregulation and IL-6 and Tissue Factor (TF) release [63, 64]. In fact, most circulating soluble CD154 in human plasma is generated from activated platelets, and soluble CD154 levels may be an indicator of the degree of platelet activation within the host [64].

Several biologically active molecules stored in intracellular granules can be released into circulation or translocated to the platelet surface to mediate other nonhemostatic functions. Platelets have three major types of storage granules: *α*-granules, dense granules, and lysosomes. *α*-granules are the most abundant type of granule, with 40–80 per platelet, and they derive their protein content by a combination of endocytosis and biosynthesis. The proteins housed in *α*-granules include coagulation factors, chemokines, adhesive proteins, mitogenic factors, and angiogenic regulators. Studies have shown that platelets contain heterogeneous populations of *α*-granules that undergo differential patterns of release during platelet activation [65, 66]. In fact, Sehgal and Storrie have identified two classes of *α*-granules: one that contains fibrinogen and another that contains von Willebrand factor (vWF) [67]. Therefore, it is likely that there are distinct granule subpopulations with differentially packaged immunomodulatory substances in a specific manner to respond to different types of tissue damage.

Platelets contain several preformed molecules necessary to mediate hemostasis. In addition, platelets contain large amounts of mRNA, and the translational machinery packaged during platelet formation can synthesize proteins during hemostatic and inflammatory events [68–71]. Following thrombin activation, proteomic analyses have demonstrated that platelets secrete more than 300 different proteins, such as interleukin-1 (IL-1), Toll-like receptors (TLRs), and CD154, which are clearly involved in host defense processes. Platelet CD154 expression may have an important role in linking the innate and adaptive immune responses and promoting protective immunity [72].

It has also been suggested that platelets act as key effector cells in inflammation and the immune continuum [73, 74]. Platelets store and release many biologically active substances, including growth factors, cytokines, and chemokines. Their impact on immune cells is associated with the induction of leukocytes and progenitor cells to the site of pathogen permeation or vascular injury inflow, as well as endothelial cells. Platelets interact with neutrophils, monocytes, and lymphocytes to activate them and they also form platelet-leukocyte aggregates that immobilize pathogens and prevent their spreading. Furthermore, platelets can absorb pathogens to target the immune response against them. It is also assumed that the presence of surface receptors such as TLRs affects their initiation and activity in the immunological response [75].

Platelets express three (hem) immunoreceptor tyrosine-based activation motif- (ITAM-) coupled receptors: glycoprotein (GP) VI, a receptor for collagen and laminin in the extracellular matrix that signals via the associated ITAM-containing Fc receptor *γ* chain (FcR*γ*), Fc*γ*RIIA, an ITAM-containing receptor for immune complexes, and C-type lectin-like receptor- (CLEC-) 2, a hemITAM-containing podoplanin receptor expressed on select cell types such as podocytes, lymphatic endothelial cells, and type I alveolar cells [76, 77]. The majority of studies on platelet ITAM signaling have focused on the role of GPVI/FcR*γ* in hemostasis and thrombosis at sites of vascular injury or plaque rupture. Platelets may be activated by different agonists, including those that recognize G protein coupled receptors (GPCRs) and soluble ligands such as thrombin and ADP. Moreover, immunoreceptors such as GPVI trigger outside-in signals to human platelets that result in rapid and, in some cases, sustained functional responses. These processes lead to the expression of several cytokines, chemokines, and cell surface molecules that initiate and perpetuate hemostasis and also alert the immune system and induce leukocyte recruitment to the injured tissue [60]. FC*γ*RIIA is best known for its role in immune-mediated thrombocytopenia and thrombosis. Recently, authors identified a critical role for GPVI and CLEC-2 in vascular integrity maintenance at sites of inflammation [78].

## 4. Hemostasis

Hemostasis is a dynamic process regulated by several mechanisms to prevent bleeding and includes two processes: (1) primary hemostasis involving vascular constriction, platelet activation, and aggregation; (2) secondary hemostasis involving the activation of coagulation mechanisms, clot formation, and its subsequent dissolution by fibrinolysis. Blood coagulation is initiated by exposure to membrane-bound Tissue Factor (TF), which is constitutively expressed on the surface of cells surrounding the vasculature (fibroblasts and muscle cells), to form a hemostatic envelope that prevents excessive bleeding after vascular injury [79–81]. Monocytes and endothelial cells do not express TF but express it during pathological conditions [82, 83] and upon exposure to inflammatory cytokines such as TNF-*α* and IL1-*β* [84].

TF is the cellular receptor and cofactor for plasma factor VIIa. The complex TF-VIIa catalyzes the conversion of factor X to Xa, which further assembles into the prothrombinase complex formed by factor Xa, factor Va, factor II (prothrombin), and calcium, thereby generating thrombin. In turn, thrombin converts fibrinogen into fibrin. The TF-VIIa complex can also activate factor IX, leading to assembly of the intrinsic tenase complex formed by factor IXa, factor VIIIa, factor X, and Ca^+2^, which generates additional factor Xa to form an amplification loop [80]. In addition to its procoagulant role, TF exerts proinflammatory activity by activating membrane receptors sensitive to coagulation proteases, such as factor VIIa, factor Xa, and thrombin. These receptors known as PARs (Protease Activated Receptors) are expressed in various tissues, including endothelial cells, mononuclear leukocytes [85], platelets, fibroblasts, smooth muscle cells, and others [86, 87]. PARs comprise a family of receptors (PAR1, PAR2, PAR3, and PAR4) that are uniquely activated by the proteolytic cleavage of their extracellular portion. This cleavage unmasks a new N-terminus, which serves as a tethered ligand that binds to its second extracellular domain, resulting in a variety of cellular responses. PAR1 can be cleaved and activated by thrombin, factor Xa, plasmin, activated protein C, and matrix metalloproteinase 1 (MMP1). PAR2 can be activated by factor VIIa, factor Xa, tryptase, and trypsin, but not thrombin. PARs are involved in several physiological and pathological processes and are considered to be a crucial link between coagulation and inflammation [86]. PAR1 activation may lead to multiple signaling pathway activation, including activation of PI3 kinase, Src family tyrosine kinases, and the ERK pathway and MAP kinases. PAR2 activation promotes the release of inositol triphosphate (IP3) and diacylglycerol (DAG) and subsequent increase of intracellular calcium [88]. Thus, several pathways can be activated, such as protein kinase C and ERK cascade of kinases and MAP. The TF-factor VIIa complex activates PAR2 to promote an increased inflammatory response in macrophages (production of reactive oxygen species, expression of adhesion molecules, and proinflammatory cytokines) and neutrophil infiltration. In addition, the ternary TF-factor VIIa-factor Xa complex can activate PAR1 and PAR2, potentially enhancing the inflammatory response [89].

The release of cytokines such as TNF-*α* and IL-6 can lead to activation of the coagulation cascade by the TF pathway [90, 91]. In turn, increased coagulation enzyme production may activate PAR receptors to increase proinflammatory cytokines and leukocyte migration to the infection site. PAR activation is accompanied by adhesion molecule upregulation and proinflammatory cytokine production (e.g., TNF-*α*, IL-1*β*, and IL-6) [91]. Cytokines bind to specific receptors and, together with coagulation enzymes, perpetuate the inflammatory response, which promotes increased interaction of activated monocytes, activated platelets, and endothelial cells [81]. The result is the convergence of signals leading to exacerbated TF expression to sustain coagulation. Therefore, the processes of coagulation and inflammation are closely related, and coagulation may affect inflammation, which subsequently modulates coagulation. This bidirectional relationship is mediated by PAR activation [88, 91, 92].

Three main anticoagulant pathways regulate the coagulation reaction: (1) the protein C system, (2) antithrombin (AT), and (3) Tissue Factor Pathway Inhibitor (TFPI). The protein C pathway modulates both the inflammatory and hemostatic systems [93] and is composed of four main constituents: protein C, endothelial protein C receptor (EPCR), protein S, and thrombomodulin. Protein C is proteolytically activated by thrombomodulin-bound thrombin on the endothelial cell surface upon EPCR binding [94–96]. Activated protein C (APC) acts with its cofactor protein S to proteolytically degrade the essential coagulation cofactors Va and VIIIa [97]. Antithrombin belongs to the serpin family and is an inhibitor of thrombin, factor IXa, and factor Xa. Notably, the rate of enzyme inhibition by antithrombin increases in the presence of heparin [98]. TFPI is a protease inhibitor that regulates the TF dependent pathway of blood coagulation, as its primary TF/factor VIIa complex inhibitor [99]. TFPI acts in a two-step manner. In the first step, TFPI inactivates factor Xa to form a TFPI/Xa complex; TFPI then inactivates TF-bound factor VIIa. Because TFPI/factor Xa complex formation is a prerequisite for efficient factor VIIa inactivation, the system ensures that some factor Xa generation occurs before the factor VIIa-mediated initiation of the coagulation system is inhibited.

Vascular damage is an expected effect of injury and inflammation, as previously reviewed [100]. Platelets have long been recognized to support the endothelial semipermeable function [101], attributed largely to the observation that platelet activation results in the release of proangiogenic proteins and angiogenesis inhibitors as part of the negative-feedback mechanisms that limit the angiogenic process. Overall, proangiogenic molecules influence vascular cell migration and proliferation and vessel organization and stabilization [100]. The proangiogenic proteins include vascular endothelial growth factor (VEGF), hepatocyte growth factor (HGF), transforming growth factor beta (TGF-*β*), basic fibroblast growth factor (bFGF), epidermal growth factor (EGF), platelet-derived growth factors (PDGF-A, PDGF-B, or PDGF-C), other soluble cytokines (IL-8, angiopoietin, and CXC chemokine ligand-12-CXCL12), and metalloproteases MMP-1, MMP-2, and MMP-9 [102]. For instance, VEGF, also known as permeability factor, increases endothelial permeability, causing plasma protein extravasation. Moreover, growth factors, such as TGF-*β*, PDGF, brain-derived neutropic factor (BDNF), and insulin-like growth factor 1 (IGF-1), control extracellular matrix (ECM) production [103] and, in turn, trigger collagen synthesis and accumulation [104]. The bioactive mediators and adhesive proteins expressed by activated platelets facilitate homotypic interactions between platelets and heterotypic interactions between platelets and different immune cell populations. For example, activated platelets express CD62P and can promote lymphocyte rolling and adhesion on high endothelial venules [105], and activated platelets can mediate neutrophil adhesion to the endothelium and upregulate their proinflammatory functions. Furthermore, it is now known that platelet-expressed CD154 (CD40L) can interact with CD40 on endothelial cells to induce endothelial cell upregulation of intercellular adhesion molecule 1 (ICAM-1) and vascular cell adhesion molecule 1 (VCAM-1) and release of CC-chemokine ligand 2 (CCL2), thereby promoting leukocyte recruitment to inflammatory sites [106].

## 5. Bone Marrow Suppression Is a Cause of Thrombocytopenia in Dengue

Previous reports have shown that, during the early phase of disease, bone marrow displays hypocellularity and attenuation of megakaryocyte maturation [33, 107]. The precise mechanisms underlying DENV-induced bone marrow suppression during the acute phase remain unclear. However, three main factors have been suggested: (1) direct lesion of progenitor cells by DENV; (2) infected stromal cells; (3) changes in bone marrow regulation [108]. Thrombopoietin (TPO) is a cytokine that specifically regulates megakaryocytopoiesis and platelet production by activating the TPO receptor c-MPL (myeloproliferative leukemia virus oncogene) [109, 110]. Because TPO is elevated when platelet production decreases, serum TPO levels may be a useful indicator of megakaryocytopoiesis in dengue [111]. In fact, Matondang et al. showed that TPO levels significantly increased in adult DENV patients in which circulating platelets were markedly reduced and the TPO levels inversely related to the platelet counts [112].

## 6. Increased Destruction of Platelets Causes Thrombocytopenia in Dengue

Thrombocytopenia may also be due to (1) platelet consumption during ongoing coagulopathy process, (2) activation of the complement system [113], or (3) increased peripheral sequestration [32, 114]. It has been shown* in vitro* that platelets undergo increased phagocytosis by macrophages in patients with secondary DENV infections by an uncharacterized mechanism [115]. It has also been demonstrated that DENV patients develop anti-platelet antibodies of the IgM isotype [116]. Notably, antiplatelet IgM titers in patient sera were higher in DHF/DSS compared to DF. Anti-platelet antibodies cause platelet lysis, as measured using lactate dehydrogenase activity assays. In accordance with elevated IgM titers, DHF/DSS sera caused increased platelet lysis compared to DF patient sera. In addition, cytotoxicity was much higher in the presence of complement [116]. Autoantibodies against endothelial cells and blood-coagulation-related molecules have also been identified [47, 117]. In fact, molecular mimicry between platelets, endothelial cells, or blood coagulation molecules and dengue virus NS1, prM, and E proteins may explain the cross-reactivity of anti-NS1, anti-prM, or anti-E antibodies to host proteins and play a role in disease pathogenesis. Cross-reactive antibodies may cause platelet dysfunction, endothelial cell damage, coagulation defects, and macrophage activation, which may contribute to some clinical features of DHF [118].

Some studies have shown platelet activation and apoptosis in dengue-infected patients. In this way, platelet apoptosis, platelet phagocytosis, and serum TPO levels significantly increased in patients during the acute and early convalescence phases compared to levels in patients during the convalescence phase and in healthy volunteers, suggesting accelerated platelet clearance. However, this was overcome by TPO-induced enhanced thrombopoiesis in these patients [119].

Another study later confirmed that platelets from DENV-infected patients exhibited classic signs of the intrinsic pathway of apoptosis, which include increased surface PS exposure, mitochondrial depolarization, and caspase-9 and caspase-3 activation. Moreover, all of these changes were observed when platelets from healthy subjects were directly exposed to DENV* in vitro*, which may contribute to thrombocytopenia development in dengue patients [46].

## 7. Are Platelets Directly Infected by Dengue Virus?

The detection of DENV antigens on the surface and in platelet-containing immune complexes from skin biopsy specimens has been well documented [120–122]. In addition, the association of DENV with platelets* in vitro* has been reported. Reverse transcription polymerase chain reaction (RT-PCR) and electron transmission microscopy (EM) analyses have been performed in plasma and platelets from 33 hospitalized DENV-infected children [123]. Dengue viral RNA was detected in the platelets and plasma by conventional RT-PCR and EM, which confirmed the presence of dengue viral-like particles inside platelets isolated from patients. These data suggested that the presence of DENV in platelets might be associated with platelet dysfunction. However, no evidence for competent DENV replication has been demonstrated in enriched preparations of platelets from DENV-infected patients [124]. More recently, a prospective observational study using blood samples from dengue-confirmed patients, as well as rhesus monkeys (RM) experimentally infected with DENV, revealed that DENV antigen was present in small vesicles of varying size and more frequently in anucleated cells associated with platelets. DENV RNA was observed in a highly enriched CD61(+) cell population from infected RM during the acute stage. These results suggest that virus-containing CD61(+) cells are directly linked to platelet dysfunction and low platelet count characteristics of dengue patients [125].

## 8. The Balance between Coagulation, Fibrinolysis, and Anticoagulant Pathways in Dengue

Both coagulation and fibrinolysis are activated during acute dengue infection, leading to alterations in their parameters [126–129]. The kinetic profile of the circulating markers of coagulopathy, such as D-dimer (DD) [130], activated partial thromboplastin time (aPTT) [130, 131], and prothrombin time (PT) [127, 130], demonstrated that these parameters are increased in patients in the acute phase of the disease. Importantly, circulating TF levels were significantly higher during the febrile phase, especially in FHD [129] and SCD patients [127], followed by a gradual normalization during the convalescent phase. The circulating levels of the natural anticoagulants protein C, protein S, and antithrombin are significantly reduced during the early disease stages. Total TFPI levels were moderately elevated during the acute phase but not after hematocrit correction. Higher Plasminogen activator inhibitor-1 (PAI-1) levels and lower protein S levels were associated with an increased severity of bleeding [127].

Initially, evidence showed that prekallikrein, factor XII, and complement C3 levels were significantly lower in DHF patients compared to fever control patients. Notably, the lowest mean levels were observed in dengue patients with shock. However, bradykinin concentrations decreased and mean activity levels of kallikrein inhibitors did not change in dengue patients [45]. Funahara et al. showed that all DHF patients had manifestations of acute DIC, in which they detected transient prolonged aPTT and PT, decreased platelet counts, fibrinogen, prothrombin, factor VIII, plasminogen, and antithrombin activities [48, 132]. DIC is a severe acute, subacute, or chronic dysregulation of hemostatic and fibrinolytic processes occurring as a secondary complication in a variety of diseases including cancer and sepsis. The mechanisms that trigger DIC are primarily related to increased expression of the clotting initiator protein TF into circulation and endothelial injury [133]. The initiation of DIC leads to enhanced fibrin formation, platelet activation, and microthrombus deposition in microcirculation, which may contribute to systemic organ failure. Remarkably, consumption of blood coagulation factors and platelets commonly lead to paradoxical hemorrhagic disturbances due to consumption of these hemostatic factors [94, 134]. The same group later postulated that acute DIC occurring in DHF is associated with increased vascular permeability [132]. These parameters have been confirmed by another study that showed that PTT and PT act as indices in predicting bleeding and outcome in DHF, as mortality was 6-fold higher in patients with platelet counts <50,000/microliters compared to patients with platelet counts >50,000/microliters [135]. More recently, a study confirmed the predictive value of the hemogram (i.e., peripheral white cell count, platelet count), coagulation profile (i.e., PT, aPTT), and blood chemistry (i.e., alanine aminotransferase (ALT) and aspartate aminotransferase (AST)) in DF/DHF diagnosis [136].

Because hemostasis depends on the balance between coagulation and fibrinolysis, some coagulation parameters (platelet count and aPTT) and fibrinolytic parameters (tissue plasminogen activator, tPA, and PAI-1) have been evaluated in DHF/DSS and DF patients. DF patients show thrombocytopenia, aPTT prolongation, and increased tPA levels, indicating activation of coagulation and fibrinolysis. These parameters indicate more severe activation of coagulation and fibrinolysis in DHF/DSS patients. In the convalescent stage, an increase in the PAI-1 level and platelet count with a concomitant decrease in tPA level and return to normal aPTT has been reported in both DHF/DSS and DF patients. Therefore, the activation of coagulation and fibrinolysis during the acute stage of DENV infection is offset by increased platelet and PAI-1 during convalescent stage. Altogether, these results suggest that the degree of DENV infection-induced coagulation and fibrinolysis activation is associated with disease severity [137].

**Figure 1:**
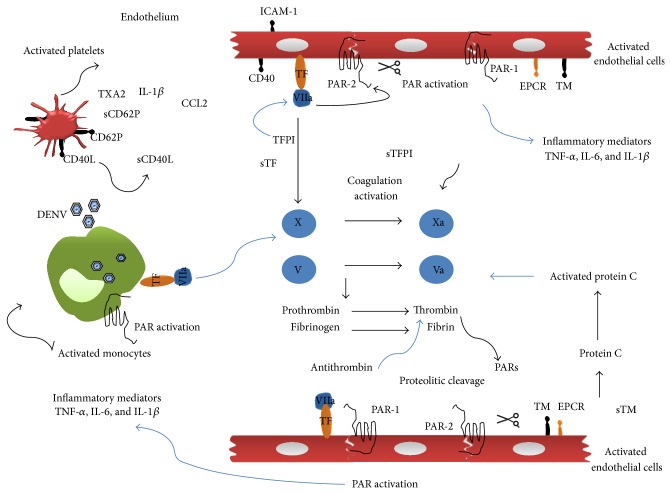
Coagulation and inflammatory response activation during dengue. DENV infection is often associated with coagulation disorders. The coagulation system is activated by the host immune response. The activation of coagulation system leads to the generation of thrombin and intracellular signaling through PAR; platelet and monocyte activation and recruitment; and increased TF expression on the endothelium and monocytes. The following anticoagulant pathways balance this system: the protein C system, antithrombin (AT), and Tissue Factor Pathway Inhibitor (TFPI). Increased CD62P expression in platelets from dengue patients indicates platelet activation and therefore favors homo- and heterotypic interactions with immune cells and endothelial cells. Additionally, CD40L (CD154) expressing platelets can interact with CD40 on endothelial cells, inducing upregulation of adhesion molecule (e.g., ICAM-1), chemokine secretion (e.g., CCL2), and leukocyte recruitment. Evidence of increased circulating TF levels in dengue patients and increased expression in monocytes from severe patients suggests its importance in disease pathogenesis. The release of inflammatory cytokines leads to activation of the coagulation cascade through the TF pathway. Coagulation factors (e.g., thrombin) activate PAR and amplify proinflammatory cytokine production (e.g., TNF-*α*, IL-1*β*, IL-6, and CCL2) and leukocyte migration to the infection site. EPCR: endothelial protein C receptor; TM: thrombomodulin.

Activation in blood coagulation and fibrinolysis are frequently observed during viral hemorrhagic fevers and sepsis. Increased TF expression has been detected in monocytes/macrophages in primates experimentally infected with Ebola virus, suggesting a role in the development of coagulation disorders during infection [138]. Remarkably, TF inhibition reduces lethality in experimental virus infection models, with reduced inflammation and coagulation processes [139]. Several studies have suggested that increased TF expression has an important role in dengue pathogenesis (Figure 1). Using primary human endothelial cells (EC) infected with DENV isolated from DHF/DSS cases, Jiang et al. showed an increase in TF mRNA expression associated with a reduction in TFPI mRNA expression [140]. Moreover, Huerta-Zepeda et al. showed that DENV upregulates PAR-1 and TF in activated endothelium [141]. These data are consistent with evidence of increased TF plasma levels in DHF DENV patients [127]. Our group further demonstrated increased TF expression on monocytes from severe dengue patients. In fact, TF monocyte surface expression was inversely correlated with platelet count [142]. Interestingly, we also found significantly higher circulating TFPI levels in severe dengue patients (unpublished data). The release of cytokines such as TNF-*α* and IL-6 can lead to activation of the coagulation cascade by the TF pathway [90, 91]. In turn, further production of coagulation enzymes may activate PAR receptors, thus amplifying the increase in proinflammatory cytokines and leukocyte migration to the infection site. PAR activation is accompanied by upregulation of adhesion molecules and production of proinflammatory cytokines (e.g., TNF-*α*, IL1-*β*, and IL-6) [91], which have reportedly been found in DENV infection [143–147]. Cytokines bind to specific receptors and, together with coagulation enzymes (and vice versa), sustain the inflammatory response, which promotes increased interaction of activated monocytes, activated platelets, and ECs. The result is a convergence of signals leading to exacerbated TF expression to sustain coagulation. Therefore, the processes of coagulation and inflammation are closely related, and this bidirectional relationship is mediated by PAR activation [91].

Moreover, levels of TNF-*α*, thrombomodulin, and vWF were significantly increased in DENV patients with and without bleeding than in healthy controls. However, plasma tPA and D-dimer levels were significantly increased in patients with bleeding. The thrombin generation test showed that patients with bleeding complications had reduced thrombin formation [148]. Other interesting data demonstrated that secreted DENV NS1 might bind to prothrombin and inhibit its activation, which in turn may contribute to aPTT prolongation and hemorrhage in DHF patients [149]. Furthermore, data have suggested that DENV-induced plasminogen cross-reactive Abs enhance plasminogen conversion to plasmin, which could contribute to hyperfibrinolysis in DHF/DSS patients [150].

## 9. Platelet Dysfunction in Dengue

A number of studies have documented platelet dysfunction in DENV infection. In this context, the suppression of platelet aggregation was demonstrated during the acute phase of DHF in both shock and nonshock patients, with a simultaneous increase in release of beta-thromboglobulin (BTG) and platelet factor 4 (PF4) from platelets into plasma [114]. Production of platelet activating factor (PAF), thromboxane B2 (TxB2), and prostaglandin D2 (PGD2) was measured in mononuclear leukocytes (MNLs) from nonimmune and previously DENV-1-infected donors when infected* in vitro* with DENV-2. The authors found that MNLs, regardless of primary or heterologous secondary exposure to DENV-2, could release significant amounts of PAF, TxB2, and PGD2. PAF but not TxB2 or PGD2 levels released by MNLs were significantly higher in those obtained from previously DENV-1-infected donors [151]. The effect of DENV-2 on the morphology and physiological activation profile was measured in normal human platelets. Their results showed that DENV-2 activates platelets with an increase in P-selectin (CD62P) expression and fibrinogen-binding property. Additionally, atomic force, scanning, and transmission electron microscopy analysis revealed typical activation-related morphological changes, such as altered platelet membrane architecture, degranulation, presence of filopodia, and dilatation of the open canalicular system, in DENV-2-exposed platelets but not in controls [152]. It is well known that nitric oxide (NO) plays a physiological role in maintaining hemostasis, regulating vasodilatation, and the aggregation, recruitment, and adhesion of platelets to the vascular endothelium [153]. Previously, high nitrite or nitrate concentrations were observed in DF patients when compared with DHF patients and controls, while there were no differences between values from DHF patients and controls [154]. Although platelets can generate NO through stimulation of NO synthase [155, 156], this same study performed experiments to determine NO production after coculture of human platelets with active and inactive forms of DENV-2. Virus-platelet interactions did not contribute to increased NO levels in the culture supernatants, suggesting that platelets are not a source of NO during DF [154]. Authors have previously shown that increased rates of L-arginine transport and the cationic amino acid l-arginine that generated NO via oxidation catalyzed by a family of nitric oxide synthase (NOS) enzymes were associated with enhanced NOS activity, elevated plasma fibrinogen levels, and reduced platelet aggregation in DENV patients [157]. Recent findings from the same group provided the first evidence of platelet l-arginine-NO pathway upregulation in DHF, despite no changes in NOS enzyme protein levels, indicating that this disease affects not only platelet production from bone marrow and its peripheral destruction but also its function [158].

## 10. Platelets as Effector Cells in DENV Infection

Acute dengue is associated with platelet activation with increased expression of activated fibrinogen receptor (*α*IIb*β*3), the lysosomal marker CD63, and the *α*-granule marker CD62P. Upon maximal platelet activation by TRAP (thrombin receptor agonist peptide-6), platelet function defects were observed with a significantly reduced (*α*IIb*β*3) and CD63 expression and reduced platelet-monocyte and platelet-neutrophil interactions [159]. Beyond its direct effector role, studies have investigated the influence of platelets on cytokine production by normal human mononuclear cells. Their findings suggest that activated platelets have anti-inflammatory properties related to the interaction between CD40L and CD40 by enhancing IL-10 production and inhibiting TNF-*α* production by monocytes [160]. In addition, authors found that interaction of monocytes with apoptotic platelets mediates IL-10 secretion through PS recognition in platelet-monocyte aggregates. Moreover, IL-10 secretion required platelet-monocyte contact but not phagocytosis, demonstrating that activated and apoptotic platelets aggregate with monocytes during dengue infection and signal specific cytokine responses that may contribute to dengue pathogenesis [161].

The TLRs belong to a family of pattern recognition receptors (PRRs) and are key players in the innate response against pathogens. These receptors are expressed in many cell types including B cells, monocytes, dendritic cells (DCs), macrophages, and neutrophils [162]. Interestingly, different groups described the expression of TLR1-9 on both human and mouse platelets. Certain platelet-expressed TLRs are functional (e.g., TLR-4) and can regulate sepsis induced thrombocytopenia and TNF-*α* production* in vivo* [163–168]. Recently, the presence of the nucleotide-binding domain leucine rich repeat containing protein (NLRP3) inflammasome was found to be associated with caspase-1 activation and synthesis of IL-1*β* in platelets activated upon DENV infection. Moreover, the authors demonstrated an increased accumulation of IL-1*β* in platelets and platelet-derived MPs from dengue patients [169].

Data published by our group showed strong evidence that increased TNF-*α* levels correlate with hemorrhagic manifestations, while increased IL-10 levels correlate with low platelet counts in a cohort of Brazilian DENV-infected patients [145]. A more recent study found that circulating TGF-*β*1 concentrations on admission were significantly lower in DHF than in controls [170]. Interestingly, platelets express the largest amount of TGF-*β* in the body [171]. Although the role of TGF-*β* in platelet-mediated hemostasis is unclear, circulating platelets appear to be important for regulating blood levels of TGF-*β*. Patients with immune thrombocytopenia have low levels of circulating TGF-*β*, but their TGF-*β* levels recover after therapy to restore normal platelet counts [172, 173].

## 11. Do Platelets Increase the Risk of Endothelium Vascular Permeability in Severe Dengue Disease?

Previous work by Butthep et al. showed that diverse cells, such as platelets, white blood cells, neutrophils, lymphocytes, and large lymphocytes, but not basophils and eosinophils, were preferentially bound to dengue-infected endothelial cells compared to control endothelial cells. It was suggested that the increased binding of platelets to endothelial cell could contribute to thrombocytopenia in DHF patients [174]. Protein disulfide isomerase (PDI), an endoplasmic reticulum protein, localizes on the platelet surface [175] and is involved in the regulation of integrin-mediated platelet aggregation, as anti-PDI Abs blocked platelet adhesion and aggregation [176]. Previous studies demonstrated that platelet membrane PDI is recognized by anti-DENV NS1 Abs and, recently, Rachman et al. observed similar kinetics of NS1 and PDI antibodies [177]. Anti-DENV NS1 reduced the PDI enzymatic activity and ADP-stimulated platelet aggregation. The DENV NS1 amino acid residues 311–330 (311–330) represent a dominant epitope with sequence homology to the thioredoxin domain of PDI [178]. In contrast, although dengue patient sera inhibited platelet aggregation, there is no correlation between NS1 antibodies and PDI antibodies with platelet aggregation dysfunction, suggesting that other mechanisms could be involved in platelet aggregation inhibition [177]. While some studies confirm the lack of association between VEGF and severity of illness in dengue virus infection, others have documented significantly higher VEGF levels in patients with DHF [179–182]. TGF-*β* showed a very significant and positive correlation with platelet counts, consistent with platelet release [183]. Angiopoietins are other key regulators of vascular integrity and are stored in platelets. Dengue-associated thrombocytopenia and endothelial activation are associated with an imbalance in angiopoietin-2: angiopoietin-1 plasma levels. In fact, the authors demonstrated that there was an inverse correlation between angiopoietin-1 and markers of plasma leakage and a positive correlation between angiopoietin-2 and markers of plasma leakage in DHF/DSS patients [184]. Hottz et al. showed that dengue-infected patients with signs of increased vascular permeability were strongly associated with a higher percentage of IL1-*β*-positive platelets and IL1-*β*-rich platelet-derived MPs, as well as caspase-1 activation compared to patients with no evidence of altered vascular barrier function. These results were confirmed when MPs from DENV-2-exposed platelets caused an increase in endothelial cell permeability that was blocked by IL-1Ra [185].

## 12. Conclusion

Thrombocytopenia, coagulopathy, and vasculopathy are hematological abnormalities related to platelet and endothelial dysfunction generally observed in severe dengue. Among the causes and consequences, previous data have suggested an imbalance between clotting* versus *fibrinolysis systems as DIC. In a minority of patients with severe or prolonged shock, the abnormalities may be profound and, in combination with severe thrombocytopenia and the secondary effects of hypoxia and acidosis, may result in true DIC and major hemorrhage. DIC is primarily triggered by TF release and may induce PAR membrane receptor activation on circulating monocytes and vascular endothelial cells in dengue patients, forming a crucial link between coagulation and inflammation. Despite limited clinically significant bleeding and only mild alterations in the results of coagulation screening tests, children with DSS had significant abnormalities in all of the major pathways of the coagulation cascade. The low circulating levels of proteins C, S, and antithrombin are likely related to leakage of these proteins through the vascular endothelium and correlate with the severity of shock. Elevated levels of TF, thrombomodulin, and PAI-1 reflect endothelial, platelet, and/or monocyte activation and may be a secondary response to direct activation of fibrinolysis by the dengue virus. Comorbidities in dengue patients result in complications leading to deaths. Another study reported that dengue patients with allergies or diabetes are 2.5 times more at risk of developing DHF. Likewise, a higher frequency of complications has been reported in dengue patients suffering from hepatitis. Hyperferritinemia was described to be associated with clinical disease severity in children, and is currently used as a laboratory marker for dengue. In fact, the coagulation and fibrinolytic systems are highly activated in dengue patients with hyperferritinemia. Furthermore, cytokines and coagulation mediators, other bioactive mediators, and adhesive proteins perpetuate the inflammatory response, promoting increased interaction between immune cells, platelets, and ECs, contributing to thrombocytopenia. Moreover, it has been suggested that thrombocytopenia arises from both decreased production of cells from bone marrow associated with an increased peripheral destruction of platelets. The cross-reactive antibodies anti-NS1, prM, and E viral proteins against platelets, endothelial cells, or coagulatory molecules may cause platelet dysfunction, endothelial cell damage, coagulation defects, and macrophage activation. Impaired platelet function could increase the risk of vascular fragility, leading to hemorrhage and contributing to plasma leakage in DHF/DSS. There are fewer studies in dengue about platelets as effector immune cells. To date, no scientific studies have suggested that platelet granule constituents can amplify inflammation and vascular permeability alteration during dengue. Several mechanisms are involved in thrombocytopenia and platelet dysfunction in dengue, indicating the complexity of dengue immunopathogenesis.
